# Supplementary material for: RUVBL1 in Clear-Cell Renal Cell Carcinoma: Unraveling Prognostic Significance and Correlation with HIF1A
Source: Cancers (Basel). 2024 Mar 25;16(7):1273. doi: 10.3390/cancers16071273 (PMC11011037; doi:10.3390/cancers16071273)
Supplement: Supplementary file 1 [file cancers-16-01273-s001.zip › cancers-2881690-supplementary.pdf]

cancers MDPI

Supplementary Materials

Supplementary Table S1. Clinicopathological characteristics of 475 patients with ccRCC from TCGA cohort.

Variables	Number (%)
**Gender**	
**Females**	163 (34.32)
**Males**	312 (65.68)
**Age**	
**≤60**	239 (50.32)
**>60**	236 (49.68)
**Grade**	
**G1**	11 (2.32)
**G2**	203 (42.74)
**G3**	189 (39.79)
**G4**	72 (15.16)
**pT status**	
**T1**	237 (49.89)
**T2**	61 (12.84)
**T3**	167 (35.16)
**T4**	10 (2.11)
**pN status**	
**Nx**	235 (49.47)
**N0**	225 (47.37)
**N1**	15 (3.16)
**Stage**	
**I**	234 (49.26)
**II**	50 (10.53)
**III**	119 (25.05)
**IV**	72 (15.16)

Supplementary Table S2. Clinicopathologicalcharacteristics of 99 patients with ccRCC from our cohort.

Variables	Number (%)
**Gender**	
**Females**	31 (31.31)
**Males**	68 (68.69)
**Age**	
**≤64**	58 (58.59)
**>64**	41 (41.41)
**Grade**	
**G1**	25 (25.25)
**G2**	64 (64.65)
**G3**	10 (10.10)
**pT status**	
**Tx**	1
**T1**	28 (28.57)

**T2**	28 (28.57)
**T3 i T4**	42 (42.86)
**pN status**	
**N0**	92 (92.93)
**N1**	7 (7.07)
